# Supplementary material for: Visualizing Sacbrood Virus of Honey Bees via Transformation and Coupling with Enhanced Green Fluorescent Protein
Source: Viruses. 2020 Feb 18;12(2):224. doi: 10.3390/v12020224 (PMC7077286; doi:10.3390/v12020224)
Supplement: Supplementary file 1 [file viruses-12-00224-s001.zip › supp/Supporting information R.docx]

**Supporting information**

**S1**

**
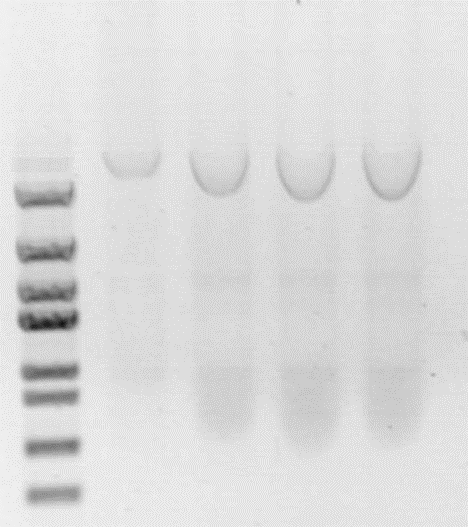
**

**Fig S1. TAE gel electrophoresis of transcribed viral RNA with an 8 kb DNA marker.** Transcribed viral were loaded into TAE gel that used for DNA gel electrophoresis for a quick exam, different amounts of the RNA were loaded in wells.


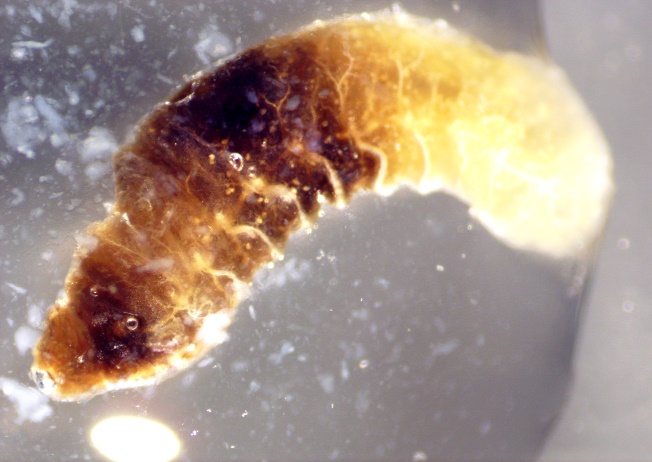

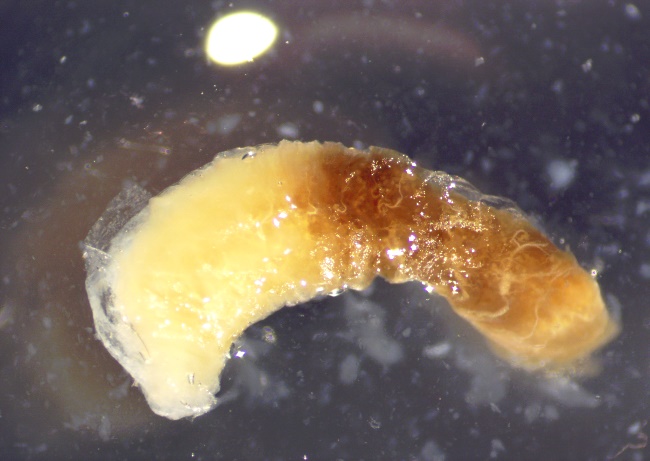


**Figure S2. Dead larvae showed SBV-like symptoms, color changes and unshed cuticle.**

**
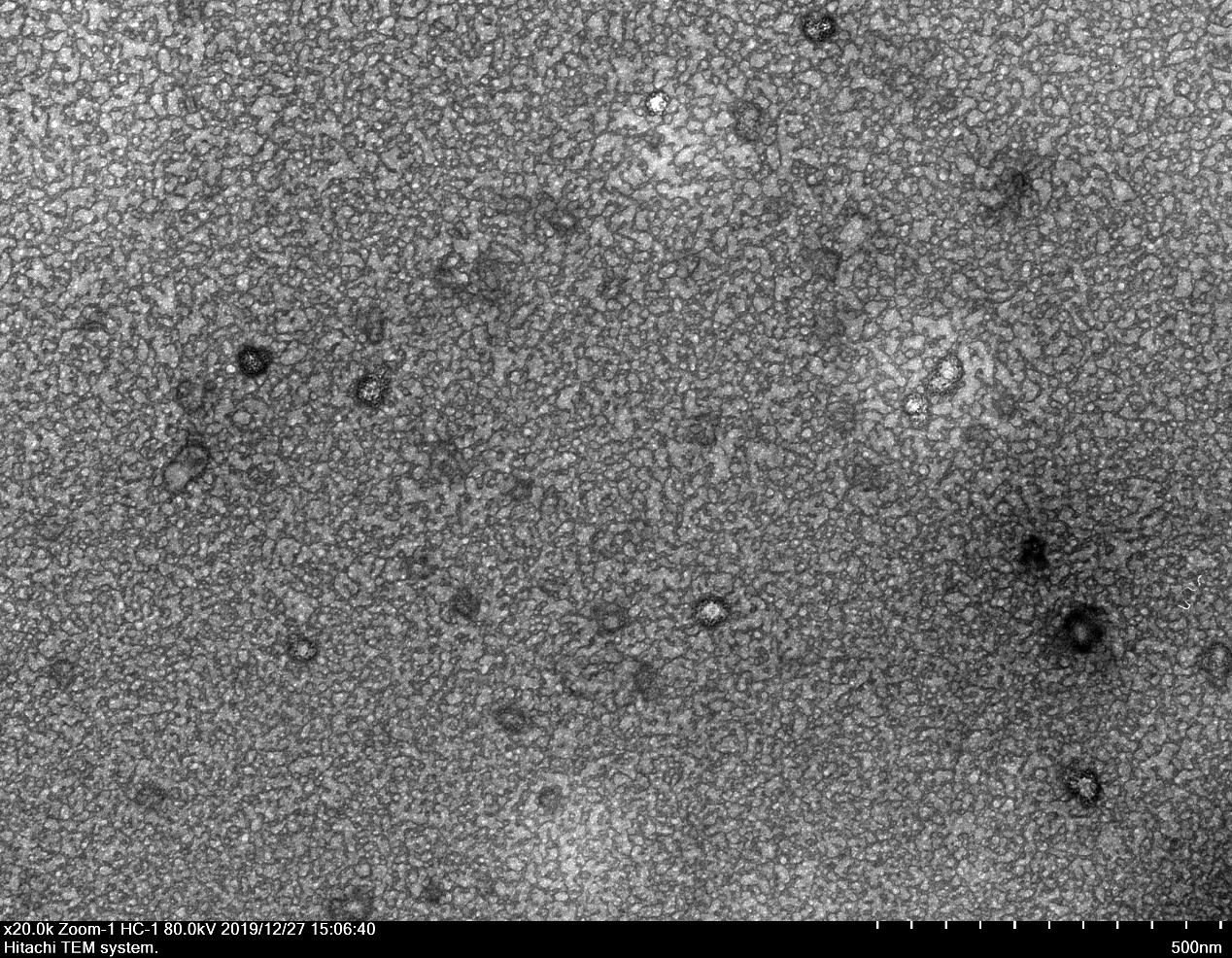
**

**Fig S3. Negative stain of produced virions under TEM.** Crude virus isolation generated from the third passage, second oral inoculation set, was diluted and then stained using regular negative stain protocol. The sample was zoomed 20,000× and observed under a Hitachi TEM system.

**
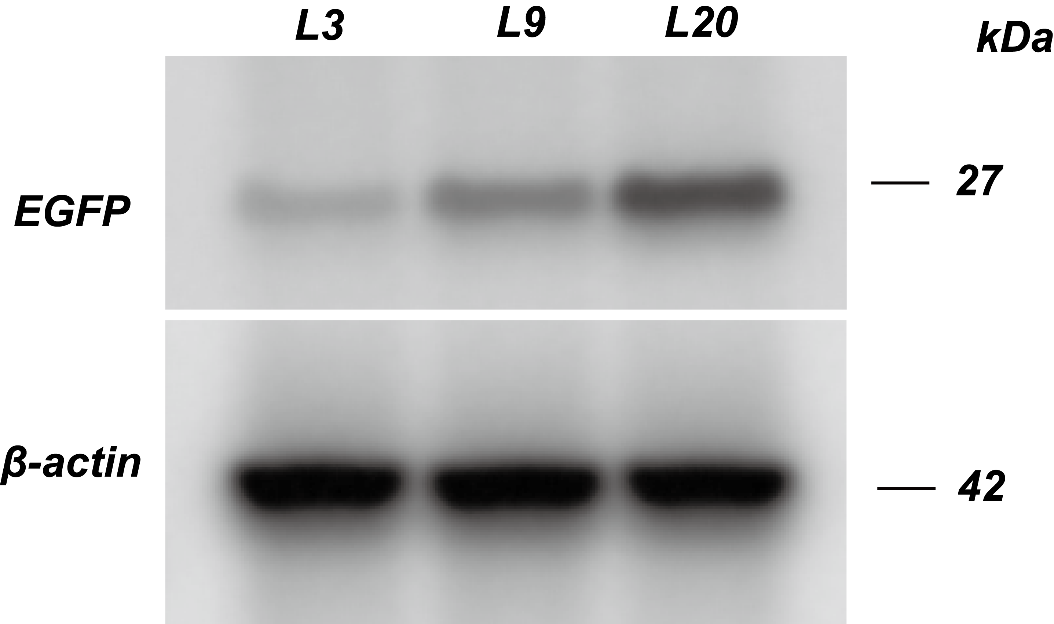
**

**Fig. S4. Western blot analysis of randomly selected infected *A. cerana* larvae, the second oral passage (P2) of the clone.** The larvae were randomly selected according to the serial number that we assigned in the trial.

S2

Table S1. Primers used in this study

| **Name** | **Sequence** | **Position*** |
| --- | --- | --- |
| T7-CSBV5' | GCTAATACGACTCACTATAGtacgaatcgtgattcgattcattatttcgcctgagta | 1-37  (in small-cap) |
| CSBV 16F | GATTCATTATTTCGCCTGAGTAA | 16 |
| CSBV 1R | CTCCGCATAACCGTATCAT | 2000 |
| CSBV 2F | GATCAGGATTACGAAGGACAA | 1735 |
| CSBV 2R | GCATGGTTACATAATAATATCAC | 4597 |
| CSBV 3F | GGAAACTCTAGCCTCTGAATT | 4310 |
| CSBV 3R | CACTAGGAACGAACTCAACAC | 6328 |
| CSBV 4F | AGAGAGTAGGTGATTCGTTTG | 6185 |
| CSBV 4R | GTAAAATGCCATATATTGATATTAATCCAC | 8800 |
| CSBV 1982F | ATGATACGGTTATGCGGAG | 1982 |
| CSBV 4561F | AGGTAATCCCCTCATTGTGATA | 4561 |
| CSBV 6308F | GTGTTGAGTTCGTTCCTAGTG | 6308 |
| CSBV3end-IRES5F | ATATGGCATTTTACACCAATGTGATC | NA |
| BQCV-IRES5 | ACCAACAATGTGATCTTGC | NA |
| BQCV-IRES3 | AGGTAAATTGTTCTGTTTAGAGA | NA |
| EGFP5-IRES3R | CCCTTGCTCACCATAGGTAAATTGTTCTGTTTAGAGA | NA |
| IRES3-EGFP5F | CAGAACAATTTACCTATGGTGAGCAAGGGCGAG | NA |
| T7t-dT-EGFP3R | GGGGTTATGCTATTTTTTTTTTTTTTTTTTTTTTTTTTTTTTTTACTTGTACAGCTCGTCCATGC | NA |
| PBR T7R | CTATAGTGAGTCGTATTAATTTCG | NA |
| PBR T7tF | TAGCATAACCCCTTGGG | NA |
| CSBV5327F | CGCAACTGGCACCTCAATCTAC | 5327 (qPCR) |
| CSBV5428R | GACTCCTACACATCGGGCACTG | 5428 (qPCR) |
| CSBV-EGFP 8576F | TTGTATCAAGCACACGGTCAT | 8576 (qPCR) |
| CSBV-EGFP 8882R | CGGTGATTCCAACAATAGCA | 8882 (qPCR) |
| CSBV3400F | TGATGCGAGGTTAGAGCAAC | 3400 |
| CSBV4420R | CAATCGTCGTAAACCACCAC | 4020 |
| EGFPF (9174F) | TCGTGACCACCCTGACCTAC | 9174 |
| EGFPR (9698R) | CGTCCATGCCGAGAGTGA | 9698 |

*Positions of the first nucleotide in the referenced SBV (CSBV) genome GenBank #KM495267 (1-8800), sequence positions after 8800 are added sequences
