# Supplementary figures and images for: Visualizing Sacbrood Virus of Honey Bees via Transformation and Coupling with Enhanced Green Fluorescent Protein
Source: Viruses. 2020 Feb 18;12(2):224. doi: 10.3390/v12020224 (PMC7077286; doi:10.3390/v12020224)

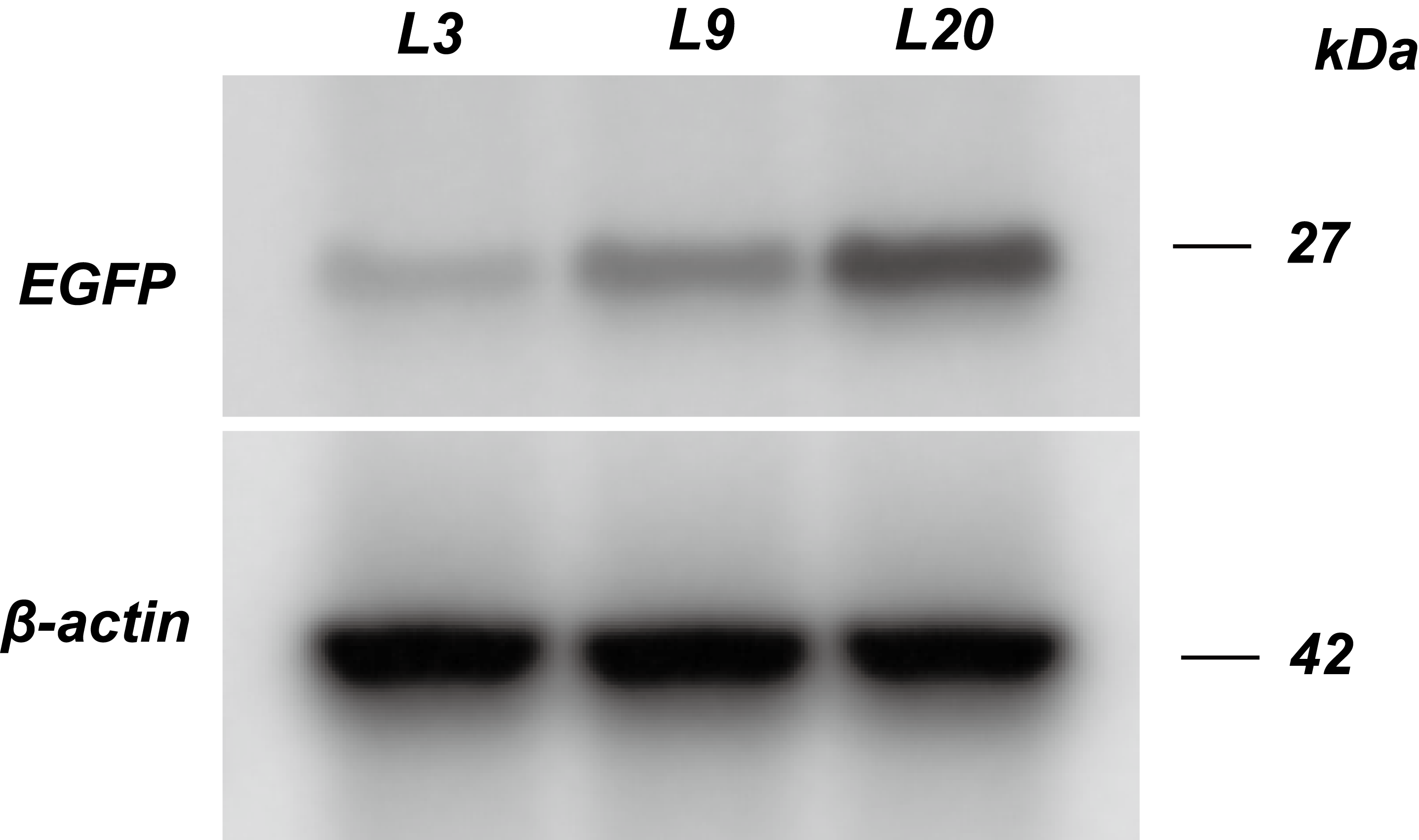

Supplement: Supplementary file 1 [file viruses-12-00224-s001.zip › supp/Fig.S4.tif]

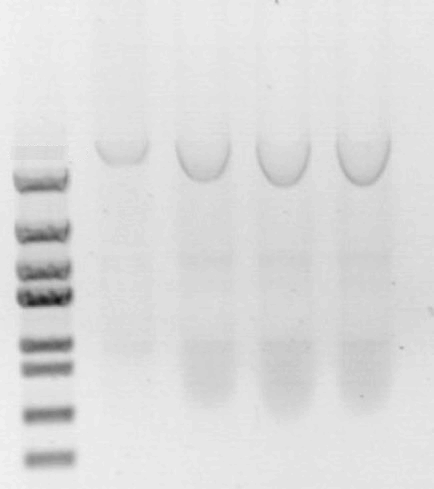

Supplement: Supplementary file 1 [file viruses-12-00224-s001.zip › supp/FigS1 gel.tif]

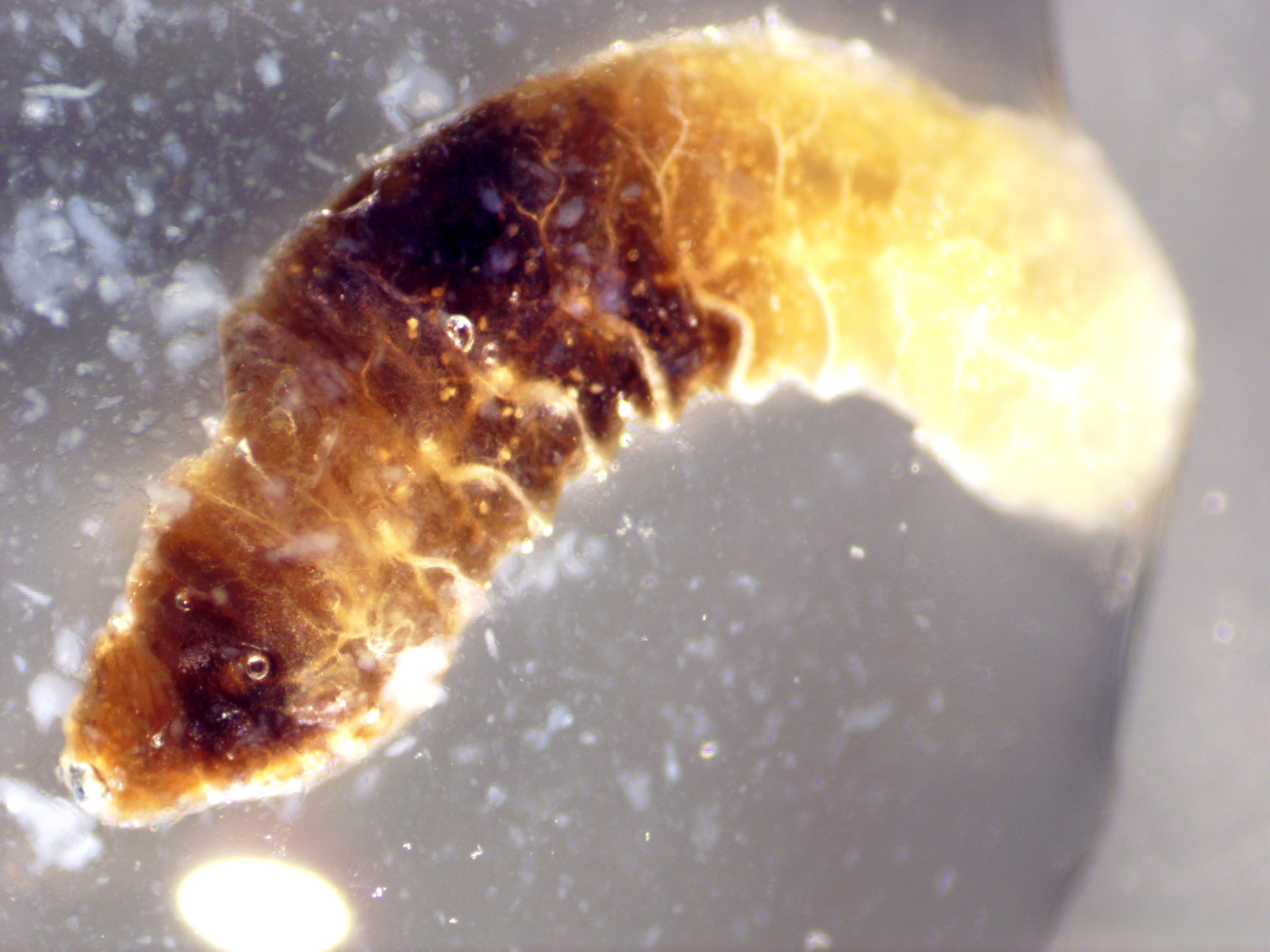

Supplement: Supplementary file 1 [file viruses-12-00224-s001.zip › supp/FigS2A.tif]

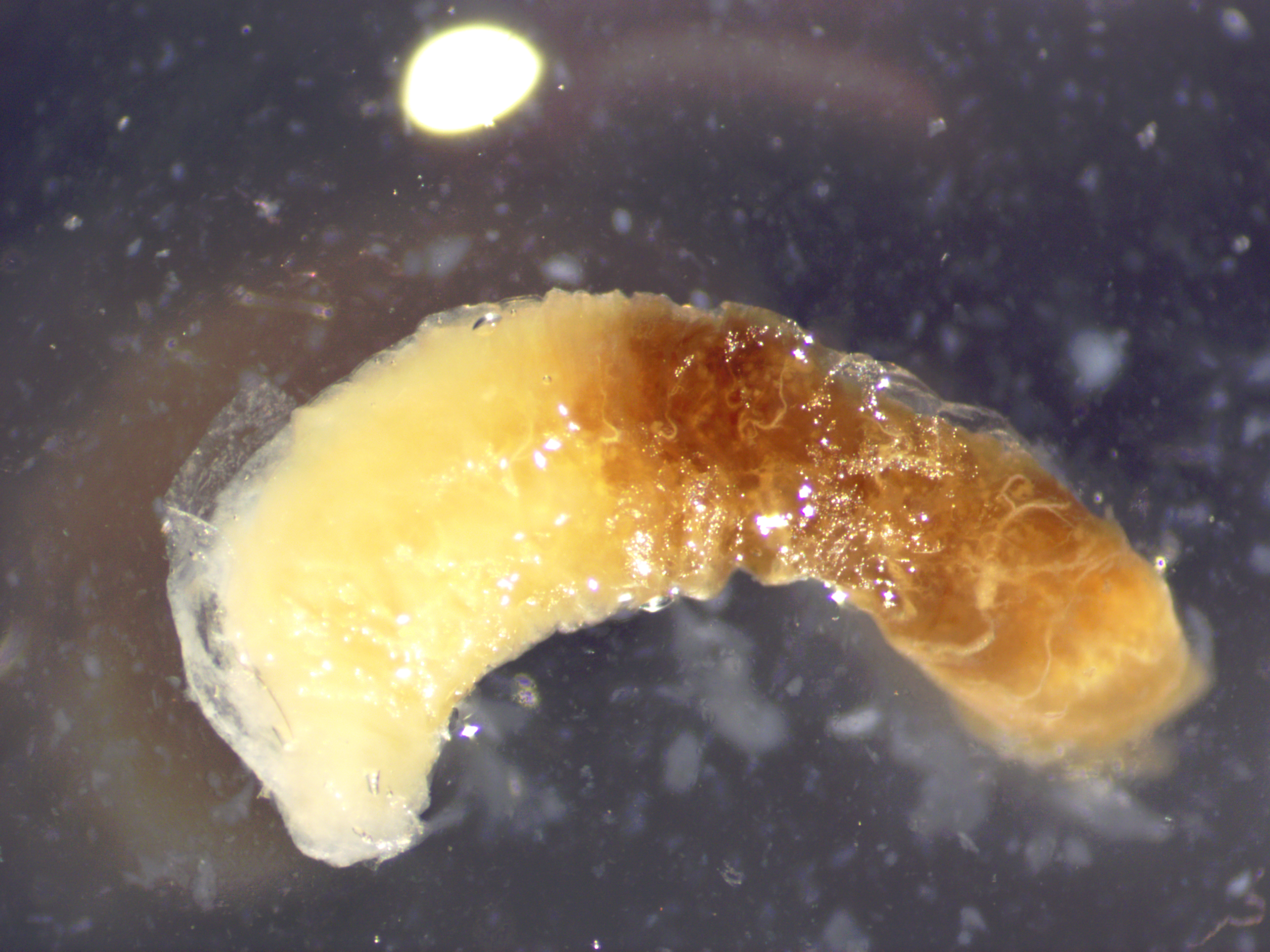

Supplement: Supplementary file 1 [file viruses-12-00224-s001.zip › supp/FigS2B.tif]

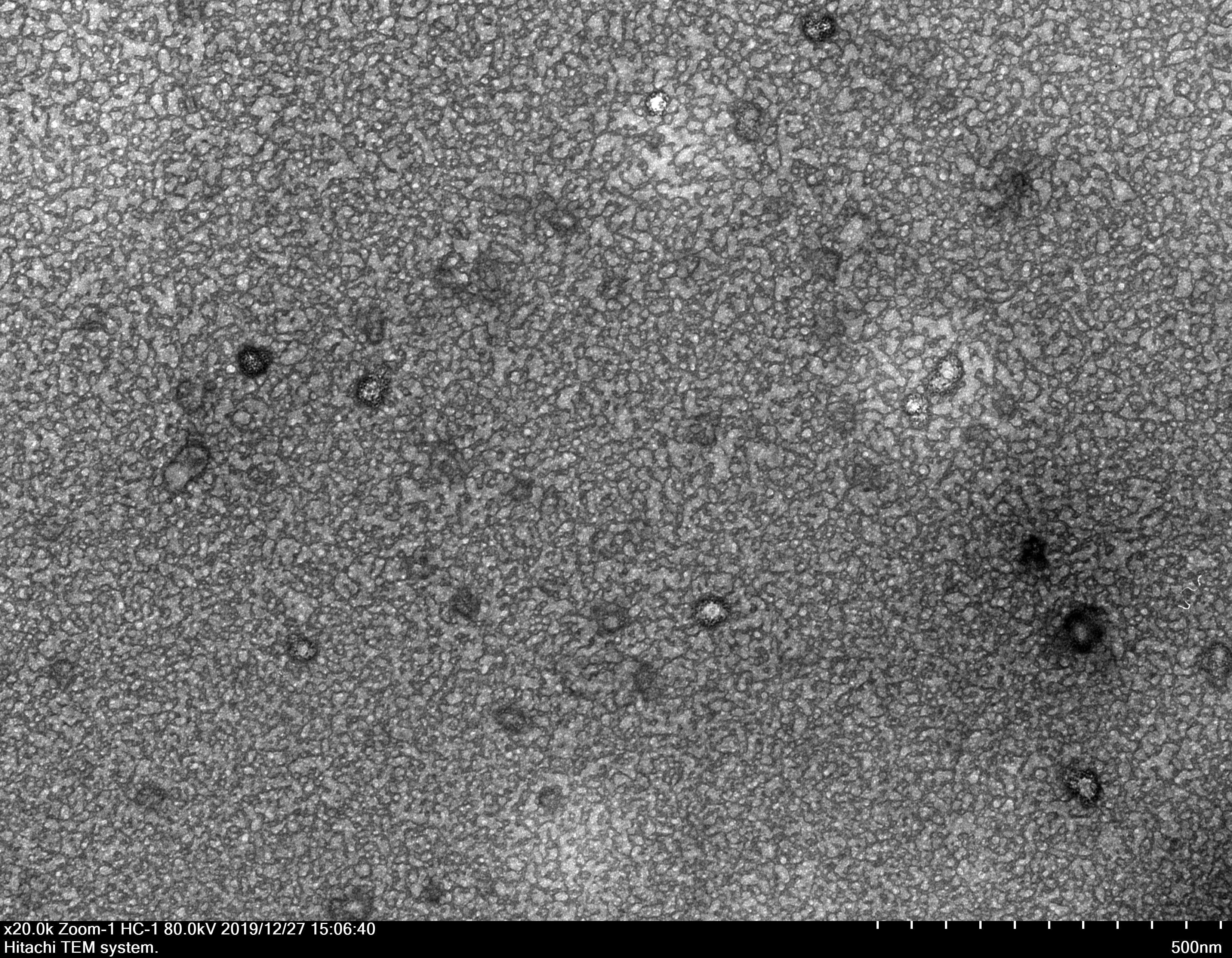

Supplement: Supplementary file 1 [file viruses-12-00224-s001.zip › supp/FigS3.tif]
